# A Multifunctional Metal–Phenolic Nanocoating on Bone Implants for Enhanced Osseointegration via Early Immunomodulation

**DOI:** 10.1002/advs.202307269

**Published:** 2024-03-06

**Authors:** Jin Liu, Yilin Shi, Yajun Zhao, Yue Liu, Xiaoru Yang, Kai Li, Weiwei Zhao, Jianmin Han, Jianhua Li, Shaohua Ge

**Affiliations:** ^1^ Department of Biomaterial & Periodontology & Implantology School and Hospital of Stomatology Cheeloo College of Medicine Shandong University & Shandong Key Laboratory of Oral Tissue Regeneration & Shandong Engineering Laboratory for Dental Materials and Oral Tissue Regeneration & Shandong Provincial Clinical Research Center for Oral Diseases Jinan 250012 China; ^2^ Central Laboratory,Peking University School and Hospital of Stomatology & National Center for Stomatology & National Clinical Research Center for Oral Diseases & National Engineering Laboratory for Digital and Material Technology of Stomatology Beijing 100081 China

**Keywords:** bone implants, metal–phenolic networks, osseointegration, osteoimmunomodulation

## Abstract

Surface modification is an important approach to improve osseointegration of the endosseous implants, however it is still desirable to develop a facile yet efficient coating strategy. Herein, a metal–phenolic network (MPN) is proposed as a multifunctional nanocoating on titanium (Ti) implants for enhanced osseointegration through early immunomodulation. With tannic acid (TA) and Sr^2+^ self‐assembled on Ti substrates, the MPN coatings provided a bioactive interface, which can facilitate the initial adhesion and recruitment of bone marrow mesenchymal stem cells (BMSCs) and polarize macrophage toward M2 phenotype. Furthermore, the TA‐Sr coatings accelerated the osteogenic differentiation of BMSCs. In vivo evaluations further confirmed the enhanced osseointegration of TA‐Sr modified implants via generating a favorable osteoimmune microenvironment. In general, these results suggest that TA‐Sr MPN nanocoating is a promising strategy for achieving better and faster osseointegration of bone implants, which can be easily utilized in future clinical applications.

## Graphical abstract

With tannic acid (TA) and Sr^2+^ self‐assembled layer‐by‐layer on titanium substrates, the metal–phenolic network (MPN) coatings provide a hydrophilic bioactive interface, which are antioxidant, affiliative and immunoregulatory. Furthermore, the TA‐Sr nanocoatings accelerate osteogenesis and enhance osseointegration around the implant through providing a regenerative environment. In general, TA‐Sr is a facile yet efficient coating strategy, which can be easily utilized in future clinical applications.

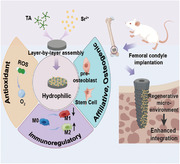

## 1 Introduction

Orthopedic implants used for joint replacements and dental implants have been the preferred alternatives for restoring the morphology and function of damaged tissues or lost teeth.^[^ ^1^, ^2^ ^]^ Titanium (Ti) and its alloys are the most commonly used materials and have been applied for decades, mainly due to their optimal biocompatibility and mechanical properties.^[^ ^3^, ^4^ ^]^ Osseointegration, which is defined as a direct structural and functional connection between organized, living bone and the surface of a load‐bearing implant, has been widely identified as an important criterion for implant success.^[^ ^5^ ^]^ Unfortunately, owing to bioinert Ti surfaces, endocrine diseases (such as diabetes mellitus, osteoporosis, and obesity), and high reactive oxygen species (ROS), the osseointegration around implants is hampered, and long‐term stability is compromised consequently.^[^ ^6^, ^7^ ^]^ In addition, orthopedic Ti implants may have irregular shapes and structures due to the complex tissue defects, it is even more challenging to achieve efficient osseointegration on irregular Ti implants.

In the past few decades, surface modification has attracted significant attention as an important method to accelerate the interaction of Ti‐based implants to promote peri‐implant bone regeneration.^[^ ^8^ ^]^ Nevertheless, surface modification of implants with complex geometries remains a challenge, especially with the rapid development of 3D printing technology.^[^ ^9^ ^]^ Conventional modification methods are grouped into two categories: physical modification and chemical modification processes. Physical methods such as plasma spraying, plasma immersion ion implantation, and magnetron sputtering are relatively easy, convenient, and efficient. However, their applications are limited due to uneven coverage on the porous surface or sophisticated structures of customized implants.^[^ ^10^ ^]^ Whereas chemical vapor deposition, wet chemical precipitation, hydrothermal synthesis, and electrochemical deposition can form homogenous layers on 3D materials with irregular shapes.^[^ ^11^, ^12^ ^]^ However, these methods suffer from low deposition rates or tedious synthetic steps, and the final coating may cause a permanent alteration of physicochemical properties, non‐degradablity, or long‐term release of cytotoxic chemical residues that will harm the long‐term success rate of implants.^[^ ^10^, ^12^, ^13^, ^14^, ^15^ ^]^

In addition, the immune response at the bone‐material interface is of great significance to implant osseointegration, which is now drawing much more attention in implant surface design and preparation.^[^ ^16^ ^]^ Osteoimmunology has revealed that immune cells interact with bone remodeling cells in complicated regulatory ways and play a key role in bone homeostasis.^[^ ^17^ ^]^ As foreign objects, implants are identified by immune cells immediately after implantation, consequently triggering a series of immune responses that affect the biological functions of cells involved in osteogenesis. A favorable host immune response during the initial stages of wound healing, i.e., early immunoregulation, is crucial for bone integration and long‐term survival of implants.^[^ ^18^ ^]^ Therefore, the design concept of advanced implants should shift from being just “immune friendly” to owning “ immunomodulatory reprogramming capability”, which is capable of regulating the local immune microenvironment and further stimulating osteogenesis and osseointegration.^[^ ^19^ ^]^ Hence, a temporary 3D coating method with the ability of osteoimmunomodulation is critical for irregularly shaped implants.

Metal–phenolic networks (MPN) are supramolecular complexes formed by the reversible chelation between metal ions and polyphenols,^[^ ^20^, ^21^, ^22^ ^]^ which have been widely used as a facile, versatile, and efficient strategy for constructing bioactive nanostructured interfaces for various biomedical applications.^[^ ^23^, ^24^, ^25^ ^]^ Combining the specific effect of both metal ions and phenolic ligands, MPN exhibits universal adhesion, tunable hydrophilicity, well‐defined composition, stimuli responsiveness, and immunomodulation, along with other beneficial properties.^[^ ^26^, ^27^ ^]^ Tannic acid (TA), a natural polyphenol extensively existing in plants, generally certified safe by Food and Drug Administration, and commonly used as a food additive, is capable of coordinating with ≈20 different metal ions (e.g., Fe, Cu, Mg, Zn, Ce, Al, Sr, etc.) to generate robust MPN films.^[^ ^28^, ^29^ ^]^ In particular, Sr is one of the trace metals in the human body. The elements of group IIA (alkaline‐earth metal) of the periodic system, to which Sr belongs together with Ca and Mg, possess similar chemical and physiological behavior, form divalent cations, and bind with protein to varying degrees in biological fluids like serum or plasma.^[^ ^30^, ^31^ ^]^ Reports showed that Sr^2+^ depressed bone resorption and simultaneously stimulated bone formation in vivo and enhanced osteoblast activity and differentiation while decreasing osteoclast formation, osteoclastogenesis‐related gene expression,^[^ ^32^ ^]^ and pro‐inflammatory cytokine expression in bone‐marrow‐derived macrophages in vitro.^[^ ^33^, ^34^ ^]^ However, current methods of introducing Sr to Ti implants reported in most literatures involve the use of bulk doping, chemical precipitation, and hydrothermal treatment,^[^ ^35^, ^36^ ^]^ which are either cumbersome or may lead to safety concerns due to long‐term release. Steffi C et al. introduced TA‐Sr‐based MPN on a Ti alloy surface and evaluated its effects on osteoblasts and osteoclasts preliminarily.^[^ ^29^ ^]^ However, the preparation process requires at least 48 h, which is not conducive to clinical application, and a facile construction approach is urgently needed. Then a few studies reported polyphenol‐Sr modified membranes or scaffolds, investigating their effect on the bone regeneration process or osteoarthritis.^[^ ^37^, ^38^ ^]^ The influence on the interfacial osseointegration around the implant surface remains unclear. Considering that MPN is biosafe and biodegradable in the physiological microenvironment, it is expected that TA‐Sr‐based MPN can offer an osteoimmunomodulation biointerface to improve the early integration of Ti implants.

In this study, we used TA and Sr^2+^ as building blocks to construct a multifunctional coating on Ti implants, with the aim of promoting osseointegration via early immunomodulation. The coordinated Sr^2+^ and TA would synergistically generate a favorable osteoimmune microenvironment to accelerate the osteogenesis process. In addition to physicochemical properties, we investigated the cellular behaviors of stem cells and macrophages on the TA‐Sr‐coated Ti, including adhesion, proliferation, differentiation, polarization, and radical scavenging. Moreover, in vivo experiments were performed to further confirm that TA‐Sr coatings would enhance osteoimmunomodulation and osseointegration at the bone‐implant interface.

## 2 Results and Discussion

### 2.1 Preparation and Characterization of the TA‐Sr MPN on Ti Plates

**Figure 1:**
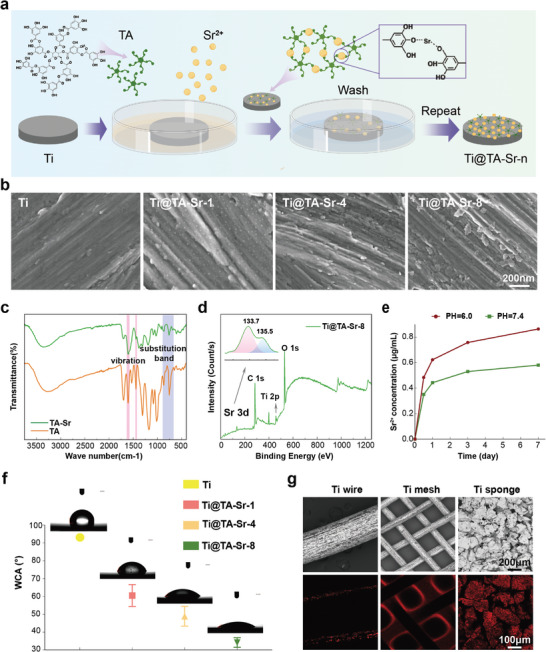
a) Schematic illustration of the stepwise assembly of MPN on Ti plates. b) Surface morphology of different samples observed by SEM. c) FTIR spectra of TA and TA‐Sr. d) XPS spectra of Ti@TA‐Sr‐8. Survey scan and Sr 3d high‐resolution spectrum (inserted). e) The cumulative release profile of Sr^2+^ release from Ti@TA‐Sr‐8 in PBS at pH 7.4 and pH 6.0. f) WCA and corresponding photos of droplets on various samples. g) Representative CLSM photos of MPN coatings on different Ti substrates with the corresponding SEM photos. The error bar represented mean ± SD (n = 5).

MPN coatings were self‐assembled layer‐by‐layer on the surface of Ti substrates by repeated incubation of Ti in TA and Sr^2+^ solutions (**Figure** **1**a). TA containing catechol and galloyl groups can be easily chelated with various metal ions and is also capable of interacting with a wide range of bioactive substances and adhering to both hydrophilic and hydrophobic surfaces due to its polyhydroxy structure as a hydrogen bond acceptor and donor.^[^ ^39^ ^]^ The reaction pH was vital for the stability of TA‐Sr during the process.^[^ ^40^ ^]^ The coordination bonds between TA and Sr^2+^ can rarely be maintained in an acidic environment, so the mixtures remained transparent after mixing TA and SrCl_2_ solutions. Once the pH was adjusted to alkalescence, the solution immediately turned silver‐white and colloidal (Figure S1, Supporting Information). The surface morphologies of TA‐Sr coated Ti plates were analyzed by scanning electron microscopy (SEM). As shown in Figure 1b, TA‐Sr was formed as granules in morphology and relatively uniform in distribution on Ti@TA‐Sr‐1. With the increase in coating layers, the particles gradually clumped together and the size increased. Surface nanoroughness and thickness of the multilayer coated Ti substrates were characterized by atomic force microscopy (AFM). As shown in Figure S2a (Supporting Information), the TA‐Sr coated Ti foils exhibited an increase in surface roughness compared to the bare Ti substrate. Specifically, Ti@TA‐Sr‐8 displayed the most significant increase in surface roughness, with the average roughness (Ra) values rising from 6.58 ± 0.50 to 10.22 ± 0.86 nm. The thickness of TA‐Sr‐8 was determined to be ≈100 nm by scratch test (Figure S2b, Supporting Information), suggesting that the average thickness of each layer is ≈13 nm, which is aligned with previous reports on MPN thickness.^[^ ^41^, ^42^ ^]^ As shown in Figure 1c, TA‐Sr MPN was characterized by a Fourier transform infrared spectrometer (FTIR). The absorption peak centered at 1600 cm^−1^ was attributed to the stretching vibration of the symmetric phenyl ring while the peak of substitution bands between 750 and 900 cm^−1^ corresponds to the symmetric out‐of‐plane bending vibration of the ring hydrogens, demonstrating that TA was successfully deposited. To further detect the elemental composition, energy dispersive spectroscopy (EDS) mapping analysis was also performed, as presented in Table S3 (Supporting Information), Sr was observed on the surface of each TA‐Sr coated Ti plate, and the element weight concentration increased layer by layer. From the survey scan spectra of X‐ray photoelectron spectroscopy (XPS), as illustrated in Figure 1d, Ti@TA‐Sr‐8 exhibited prominent carbon and oxygen peaks as the main elements, the signals of Sr elements were detected as well, and the divalent feature of Sr ions was confirmed in the high‐resolution spectra, further suggesting the successful chelation between TA and Sr^2+^ on the Ti substrates. Nanoindentation was used to evaluate the mechanical properties of Ti substrate before and after MPN coating, we utilized the unloading curves of an indentation test to extract the hardness and reduced modulus (Er) of the material. As exhibited in Figure S2c,d (Supporting Information), there was no significant difference between the mechanical properties of uncoated and coated Ti plates, suggesting that MPN coating has little influence on the mechanical properties of Ti.

The results of the ion release test in different pH buffers conducted by inductively coupled plasma mass spectrometer (ICP‐ MS) are depicted in Figure 1e. Sr^2+^ reached its initial peak concentration with a short burst release on the first day and then slowly and steadily released on the following 6 days when the pH was 7.4. However, the release performance under faintly acidic conditions (pH 6.0) mimicking the inflammatory environment changed, which is significantly faster. This phenomenon may be mainly attributed to the fact that the coordinative bonds of MPNs are generally pH‐responsive,^[^ ^26^ ^]^ which means that TA‐Sr would dissociate in an acidic environment, imparting the inflammation‐responsive capacity to TA‐Sr modified implants. The quantitative analysis of Sr^2+^ loading (Figure S3, Supporting Information) was carried out by completely digesting the coating with hydrochloric acid (HCl, pH 3.0). The total amount was as follows: Ti@TA‐Sr‐1, 0.096 ± 0.003 mg cm^−2^; Ti@TA‐Sr‐4, 0.226 ± 0.008 mg cm^−2^; and Ti@TA‐Sr‐8, 0.279 ± 0.009 mg cm^−2^, while the normal dietary intake ranges from 2 to 4 mg Sr per day and the typical Sr^2+^ concentration in human blood is between 10.57 and 12.23 mg L^−1^.^[^ ^30^ ^]^ Collectively, TA‐Sr was biocompatible and biosafe in terms of Sr loading.

The surface hydrophilicity of the Ti plates was tested by measuring water contact angle (WCA) (Figure 1f). The pristine Ti surface was hydrophobic (WCA = 93.6° ± 1.3°), while the WCA value of TA‐Sr coated Ti plate decreased dramatically, and Ti@TA‐Sr‐8 displayed the best hydrophilicity (WCA = 34.1° ± 2.9°), which was mainly attributed to the inherent superior hydrophilicity of TA molecules and the increase in surface nanoroughess. This roughness likely promotes the exposure of more hydrophilic polyphenolic groups, leading to the observed increase in WCA. This conclusion is supported by the Wenzel model, which correlates increased roughness on hydrophilic surfaces with enhanced wettability.^[^ ^43^, ^44^ ^]^ Researchers discovered that increased surface wettability had an enhanced immunomodulatory effect.^[^ ^45^, ^46^ ^]^ A wettable Ti surface is more conducive to blood clot formation and platelet activation, simultaneously reduces the number of inflammatory cells, and ulteriorly affects the cellular mechanisms involved in faster and improved osseointegration.^[^ ^47^, ^48^ ^]^ Hydrophilic surfaces also showed great potential in facilitating the initial adhesion and subsequent biological behavior of both osteoblasts and immune cells.^[^ ^49^, ^50^, ^51^ ^]^ In addition, as exhibited in Figure 1g, TA‐Sr MPN was capable of forming homogeneous coverage on the surface of different substrates with 3D complex structures including wire, mesh, and porous sponge. With such excellent surface adaptability, MPN will be fitted on intricate surfaces with various morphologies,^[^ ^41^ ^]^ suggesting that the MPN interficial assembly on Ti implants is simple yet effective Sr ions loading and coating method compatible to complex structures.

### 2.2 Radical Scavenging Capacity

**Figure 2:**
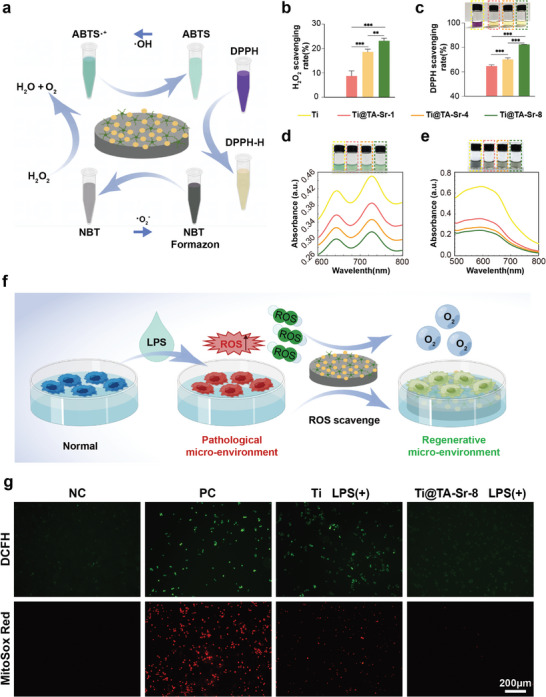
a) A schematic plot about the radical scavenging capacity of the coatings against different radicals: H_2_O_2_, O_2_^−^, ·OH, and DPPH. b) H_2_O_2_ scavenging rate of different groups (n = 5). c) DPPH scavenging rate and the corresponding digital photos (n = 5). d,e) UV–vis absorption spectra and corresponding photos of the catalyzed oxidation of ABTS (734 nm) by ·OH and NBT (560 nm) by O_2_^−^ on different groups of TA‐Sr. f) A schematic diagram of TA‐Sr reshaping the pathological micro‐environment into a regenerative one by scavenging ROS. g) Representative fluorescent images of DCFH staining for intracellular ROS and MitoSOX staining for mitochondrial ROS in macrophages incubated with LPS. The data (b,c) were analyzed by one‐way ANOVA test. The error bar represented mean ± SD, ^**^ *P* <0.01, and ^***^ *P* <0.001.

In addition to the pathological environment caused by chronic inflammation or diabetes mellitus, the invasive surgical procedures of implantation will also lead to the generation and accumulation of massive radicals, such as O_2_^−^, ·OH, H_2_O_2_·, NO·, etc., the subsequent oxidative stress will boost inflammatory reactions and compromise bone healing.^[^ ^52^, ^53^ ^]^ Nevertheless, it has been confirmed that antioxidant coatings on the surface of Ti implants significantly promoted osteogenesis surrounding the implants.^[^ ^54^, ^55^ ^]^ The antioxidant capacity of TA‐Sr was then investigated, as shown in **Figure** **2**a‐e, TA‐Sr coated Ti plates displayed their radical scavenging capacities in a layer‐dependent manner. The color of 1,1‐Diphenyl‐2‐picrylhydrazyl (DPPH), 2,2′‐Azinobis‐(3‐ethylbenzthiazoline‐6‐sulphonic acid) (ABTS) and Nitrotetrazolium Blue chloride (NBT) solution incubated with TA‐Sr modified Ti plates was lighter than that incubated with native Ti plates, and Ti@TA‐Sr‐8 exhibited the optimal radical scavenging capacity, which was indicated to be positively associated with the amount of phenolic hydroxyl. To further verify the intracellular ROS‐scavenging properties of TA‐Sr‐8 coatings (Figure 2f), lipopolysaccharide (LPS) was used to induce oxidative stress status mimicking pathological micro‐environment, and intracellular ROS was visually labeled with the fluorescent probe 2′,7′‐dichlorofluorescein diacetate (DCFH‐DA). As illustrated in Figure 2e, ROS levels were elevated in cells cultured on Ti plates after LPS treatment compared to those on TA‐Sr‐8 modified plates. Considering mitochondria as the primary source of ROS generation, mitochondrial ROS levels were evaluated ulteriorly with Red Mitochondrial Superoxide Indicator (MitoSox Red), a fluorescent dye of high selectivity. As expected, Ti@ TA‐Sr‐8 samples exhibited significantly lower fluorescence intensity than native Ti samples (Figure 2g). The above results demonstrated that TA‐Sr coatings attenuated LPS‐induced oxidative stress, thus reprogrammed the pathological micro‐environment to a regenerative one, benefiting from TA, which can scavenge ROS or enhance the intracellular antioxidant defenses under simulated oxidative stress status.^[^ ^56^, ^57^ ^]^

### 2.3 TA‐Sr Coatings Regulated Cell Morphology, Proliferation and Migration

**Figure 3:**
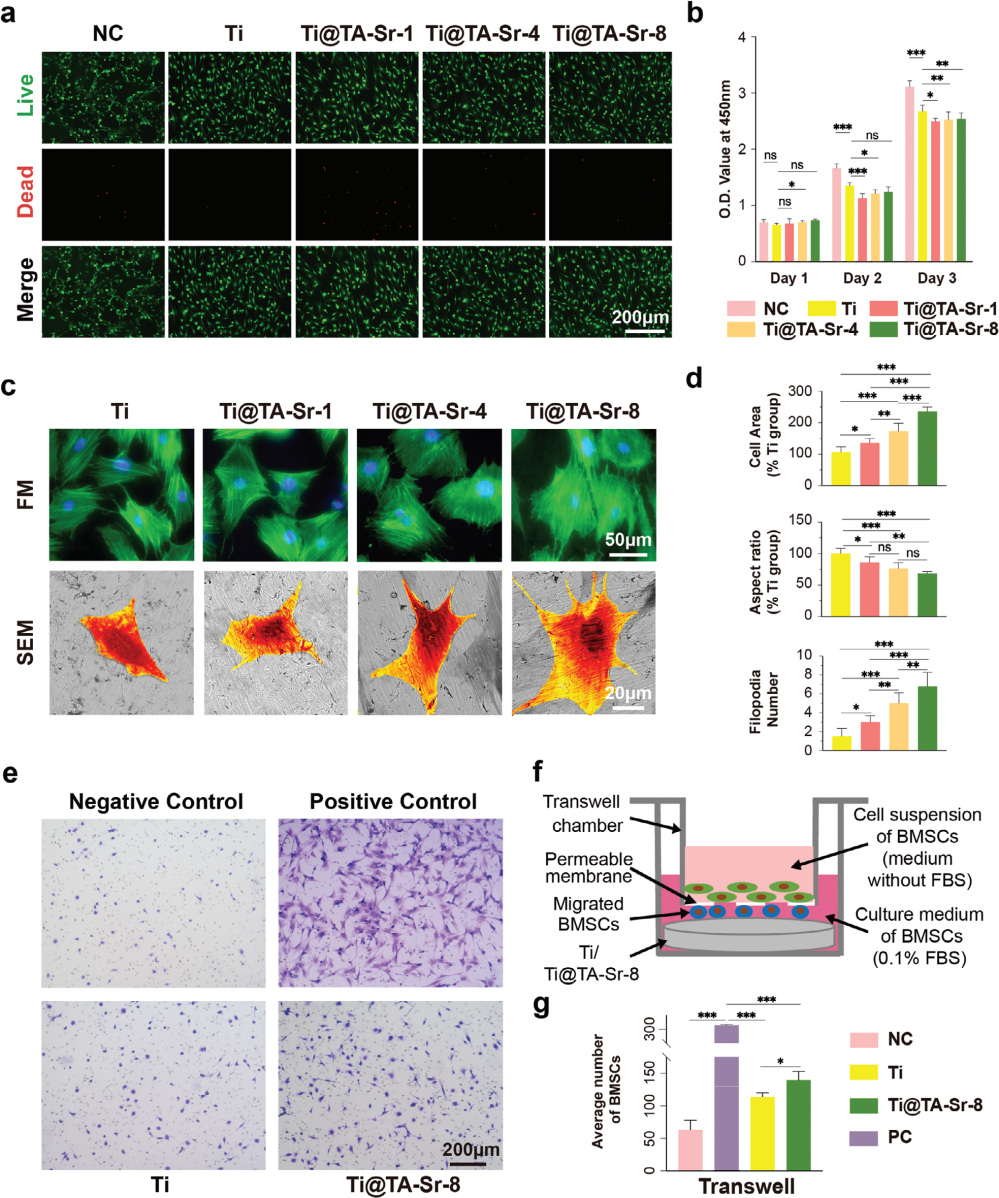
a) Live/dead staining of BMSCs after 24 h of culture (live cells, green; dead cells, red); b) Proliferation of BMSCs cultured on various samples measured by CCK‐8 (n = 6); c) Morphologies of BMSCs after 24 h of culture captured by fluorescence microscope (phalloidin‐FITC: cytoskeleton, green; DAPI: nuclei, blue) and SEM (BMSCs were pseudocolored orange for visual observation); d) Quantitative analysis of the average cell area, aspect ratio and filodopia numbers of adhesive cells according to the SEM result (n = 10); f) Schematic illustration of transwell migration assay; e) Representative pictures of BMSCs that migrated to the proximal surface toward the Ti plates (stained by crystal violet); g) Quantitative comparison of migrated cells (n = 5). The data (b,d, and g) were analyzed by one‐way ANOVA test. The error bar represented mean ± SD; ns, no significance, ^*^ *P* <0.05, ^**^ *P* < 0.01, and ^***^ *P* < 0.001.

The biocompatibility of the implant surface is necessary prior to achieving osseointegration around implants.^[^ ^58^ ^]^ **Figure** **3**a shows the viability of bone marrow mesenchymal stem cells (BMSCs) cultured on various substrates. The great majority of BMSCs were alive in all the experimental groups, indicating that each sample was non‐cytotoxic (Figure S4, Supporting Information). The cell counting Kit‐8 (CCK‐8) assay was conducted to evaluate the proliferation of BMSCs 1, 2, and 3 days after seeding. Compared to the Ti group, cell proliferation abilities of the TA‐Sr coated groups were slightly suppressed (Figure 3b), probably on account of the accelerating influence of Sr^2+^ on the differentiation of BMSCs.^[^ ^59^ ^]^

The morphologies of BMSCs cultivated on different substrates for 1 d were observed by immunofluorescent staining images (Figure 3c), where the nucleus was stained with 4',6‐diamidino‐2‐phenylindole (DAPI, blue) and the actin cytoskeleton with fluorescein isothiocyanate (FITC)‐phalloidin (green). Although BMSCs on all substrates were irregularly polygonal in shape and their cytoskeletons appeared filamentous in a regular arrangement, they showed much denser F‐actin filaments on Ti@TA‐Sr‐8. The results were further verified by SEM (Figure 3c,d). As the number of TA‐Sr coatings increased, we observed a notable enhancement in both cell area and filopodia number, coupled with a decrease in the aspect ratio. The most pronounced effect was seen with Ti@TA‐Sr‐8, which facilitated the most extensive cellular spreading characterized by the lowest aspect ratio and a maximal number of cytoplasmic projections, indicative of robust filopodial extension. The enhanced cell adhesion and spreading behaviors are closely related to the incremental increase in nanoroughness and hydrophilicity of the modified surface. Each successive layer of TA‐Sr contributed to the surface texture enhancement, thereby promoting improved cell‐material interactions. These observations are in alignment with prior studies that have established a positive correlation between surface roughness, hydrophilicity, and cellular response. Specifically, studies have shown that increased nanoscale roughness and hydrophilicity can significantly bolster cell adhesion and spreading, confirming our findings.^[^ ^49^, ^60^ ^]^ In conclusion, Ti@TA‐Sr‐8 demonstrates superior cell affinity and compatibility toward BMSCs when compared to uncoated Ti substrates.

In the process of bone regeneration, the recruitment and mobilization of endogenous stem cells is crucial in the bone remodeling stage.^[^ ^61^ ^]^ To determine the effect of TA‐Sr‐8 on stem cell recruitment, a transwell migration experiment, as shown schematically in Figure 3f, was performed. The results (Figure 3e–g) indicated that TA‐Sr‐8 promoted the migration capacity of BMSCs, which might be attributed to the synergy between TA and Sr, which have both been reported conducive to stem cell recruitment and possibly via the activation of stromal cell‐derived factor−1α/CXC chemokine receptor 4 signaling pathway.^[^ ^35^, ^62^, ^63^ ^]^ Therefore, the strategic application of TA‐Sr coatings culminates in a microenvironment that is more conducive to cell recruitment, attachment, and morphological maturation, thereby enhancing the material's potential for bone integration.

### 2.4 TA‐Sr Coatings Enhanced Osteogenesis In Vitro

**Figure 4:**
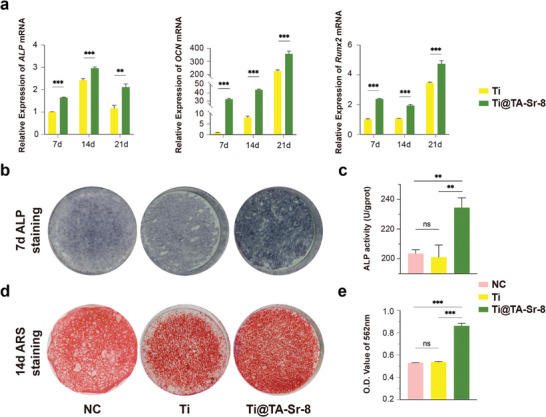
In vitro osteogenic potential of TA‐Sr coatings. a) osteogenesis‐related genes relative expression of *ALP*, *OCN*, and *Runx2* in BMSCs detected at each checkpoint by qRT‐PCR analysis (n = 3), two‐tailed unpaired Student's *t*‐test; b) Representative photographs of ALP staining of BMSCs in different groups on day 7; c) Quantitative evaluation of ALP activity (n = 3); d) Representative images and e) corresponding semiquantitative analysis of alizarin red staining of BMSCs on different substrates on day 14 (n = 4). The data (c,e) were analyzed by one‐way ANOVA test. The error bar represented mean ± SD; ns, no significance, ^*^ *P* < 0.05, ^**^ *P* < 0.01, and ^***^ *P* < 0.001.

For both animals and humans, the process of bone formation starts during the first week and is dominated by the osteogenic differentiation of stem cells to functional osteoblasts, which mainly undergo four stages: cell proliferation, extracellular matrix maturation, mineralization, and apoptosis.^[^ ^64^, ^65^, ^66^ ^]^ In this study, we first observed the expression levels of the osteogenic genes, such as alkaline phosphatase (*ALP*), osteocalcin (*OCN*), and runt‐related transcription factor 2 (*Runx2*), in BMSCs cultured and induced on different samples for 7, 14, and 21 days. As presented in **Figure** **4**a, Ti@TA‐Sr‐8 significantly enhanced osteogenic gene expression compared to pristine Ti, and it was remarkable that TA‐Sr could upregulate the early expression of *OCN* and *Runx2*, markers of advanced osteoblasts,^[^ ^67^ ^]^ and their expression levels of Ti@TA‐Sr‐8 group on day 7 were even higher than those of Ti group on day 14. The effect of TA‐Sr on extracellular matrix mineralization of BMSCs was then investigated by quantitative ALP activity assay and ALP staining. As illustrated in Figure 4b,c, there were no significant differences in the surface ALP content between the Ti and negative control (NC) groups on day 7, whereas TA‐Sr clearly boosted ALP production and activity, indicating a better potential osteogenic effect.^[^ ^68^ ^]^ Furthermore, Alizarin Red S staining (Figure 4d) of BMSCs following osteogenic induction for 14 days exhibited that the distribution of red calcium nodules on the surface of Ti@TA‐Sr‐8 was much denser than that on the surface of Ti or NC, and the corresponding semiquantitative amount of calcium content (Figure 4e) was much higher, demonstrating that TA‐Sr significantly promoted mineralization and late osteogenesis.

To understand the immediate effect of implant for osseointegration, osteoblasts (MC3T3‐E1) were also used to investigate potential osteogenic effect of Ti@TA‐Sr‐8. Compared with the Ti group, MC3T3‐E1 on Ti@TA‐Sr‐8 exhibited a much more extensive spreading morphology and smaller aspect ratio with a greater number of filament pseudopods extending from cytoplasm (Figure S5a, Supporting Information). In addition, TA‐Sr significantly upregulated osteogenic gene expression *(ALP, Collagen Type I*, and *Runx2)* and increased ALP production and activity compared to pristine Ti (Figure S5b,c, Supporting Information), indicating a better potential osteogenic effect, which is consistent with the influence on BMSCs.

### 2.5 TA‐Sr Coatings Skewed Macrophage Polarization Toward anti‐inflammatory (M2) Type

Macrophages are capable of polarizing to two distinct phenotypes in their response to changing growth conditions and new stimuli: the classically activated pro‐inflammatory (M1) and the alternatively activated anti‐inflammatory (M2) ones, and exert diverse functions in sequence by switching from a pro‐inflammatory state to a pro‐reparative state in bone regeneration.^[^ ^4^, ^69^ ^]^

**Figure 5:**
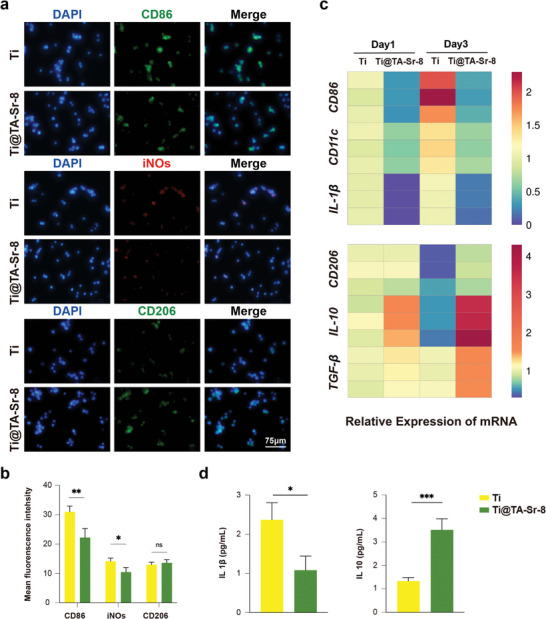
Effect on macrophage polarization of TA‐Sr coatings. a) Typical immunofluorescent images of macrophages cultured on Ti and Ti@TA‐Sr‐8 for 24 h: CD86 (M1 marker, green), iNOs (M1 marker, red), CD206 (M2 marker, green), DAPI (nucleus, blue); b) quantitative analysis of fluorescence intensity (n = 5); c) Heat map of gene relative expressions in macrophage cultured for 1 day and 3 days (n = 3); d) IL‐1*β* and IL‐10 level of macrophage on day 3 measured by ELISA (n = 3). The data (b,d) were analyzed by two‐tailed unpaired Student's *t*‐test. The error bar represented mean ± SD; ns, no significance, ^*^ *P* < 0.05, ^**^ *P* < 0.01, and ^***^ *P* < 0.001.

Although the necessary inflammatory responses after implant insertion are initiated by the presence of M1 macrophages, prolonged infiltration of M1 could lead to chronic inflammation and failure of osseointegration. On the other hand, M2 macrophages are essential for tissue reconstruction and repair by modulating and terminating the inflammatory response.^[^ ^41^, ^70^ ^]^ Consequently, endosseous implants with osteoimmunomo‐dulatory capacity would be a promising strategy to promote osseointegration. Accordingly, we studied how TA‐Sr affected the infiltration and the phenotypic transformation of macrophages. As shown with immunocytofluorescence imaging, there were fewer Cluster of Differentiation 86 (CD86)‐positive and Inducible Nitric Oxide Synthase (iNOs)‐positive (both M1 markers) macrophages infiltrated in the TA‐Sr group compared to the Ti group, whereas there was no significant difference in Mannose Receptor 1 (CD206)‐positive (M2 marker) ones between two groups on day 1 (**Figure** **5**a,b). Gene expression levels of *CD86*, Integrin alpha (*CD11c*, M1 marker) and Interleukin (*IL)‐1β*(M1 marker) were remarkably downregulated on TA‐Sr‐8 coatings on day 1, while the expression levels of the M2 phenotype markers (*IL‐10* and transforming growth factor‐beta (*TGF‐β*) were slightly upregulated on Ti@TA‐Sr‐8 and there was no difference in *CD206* (Figure 5c), which was consistent with the immunofluorescence results. The performance of TA‐Sr in manipulating macrophages was also substantiated on day 3. TA‐Sr‐8 maintained inhibiting the expression of M1 phenotype markers, and delightedly increased the expression of M2 phenotype markers compared to the Ti group. Furthermore, with the enzyme‐linked immunosorbent assay (ELISA), we confirmed a reduction in IL‐1*β* (one of the pro‐inflammatory cytokines) and an increase in IL‐10 (one of the anti‐inflammatory cytokines) in the TA‐Sr modified group on day 3 (Figure 5d). Collectively, these results revealed that TA‐Sr possessed admirable anti‐inflammatory and pro‐healing functions, where TA inhibited macrophage polarization toward the M1 phenotype mainly attributed to its antioxidant properties,^[^ ^42^ ^]^ meanwhile Sr^2+^ could promote polarization toward the M2 phenotype synchronously.^[^ ^71^ ^]^

### 2.6 TA‐Sr Coatings Augmented Osseointegration In Vivo

**Figure 6:**
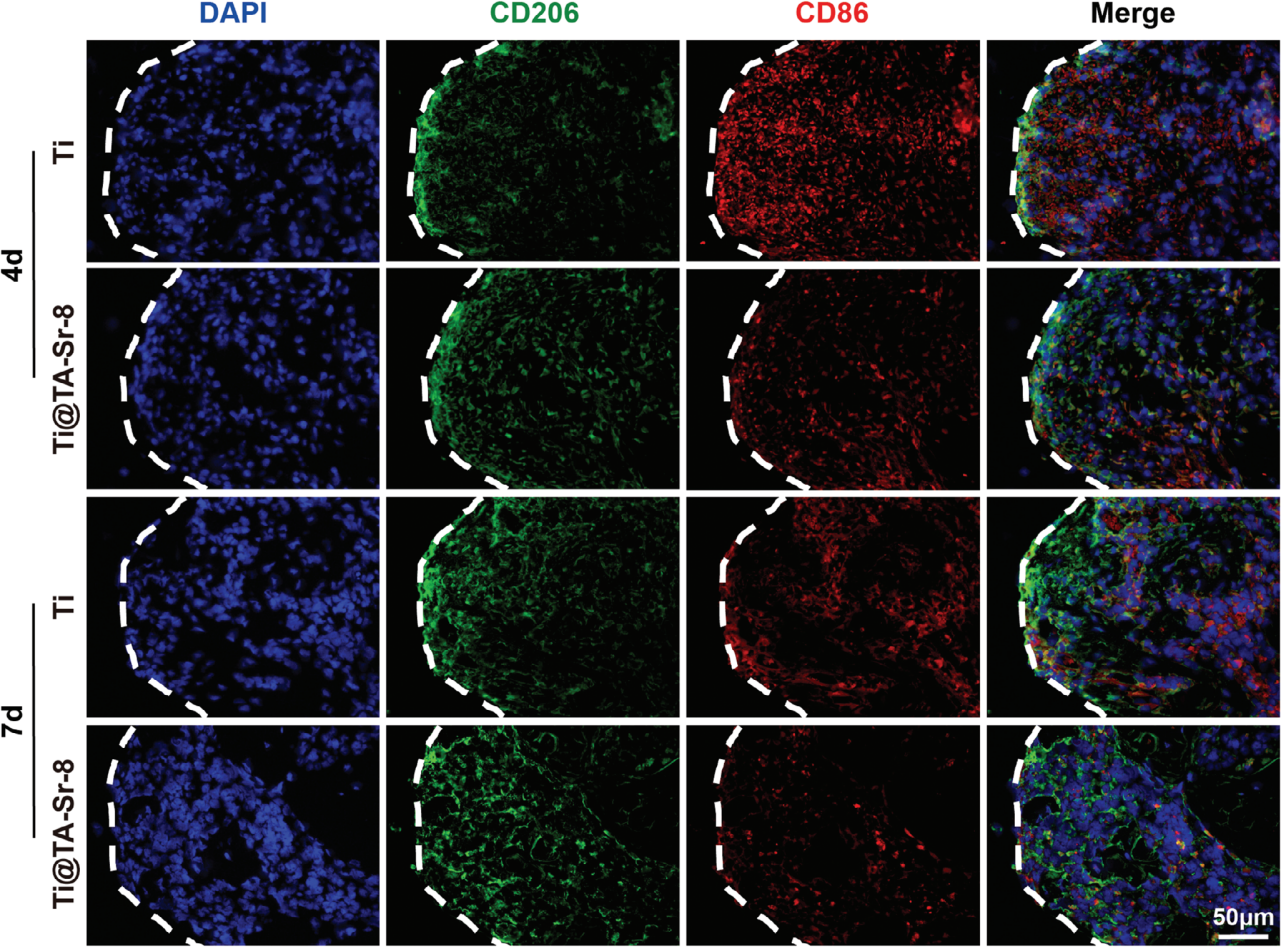
Representative fluorescent images showing the expression of CD86 (M1 marker, red), and CD206 (M2 marker, green) at the bone‐implant interface (indicated as white dashed lines) 4 and 7 days after implantation.

To further explore the effect of TA‐Sr coatings on osteoimmunomodulation and osseointegration in vivo, a rat femoral condyle implantation study was conducted. As demonstrated in **Figure** **6**, we observed a significant increase in CD206 positive cells (M2) and a decrease in CD86 (M1) positive cells at the bone‐implant interface of the TA‐Sr‐8 group 4 days after insertion, compared to the Ti group. This result was consistent with the in vitro results on the third day that TA‐Sr up‐regulated the gene expression of M2 phenotype markers while inhibiting the expression of M1 phenotype markers. 7 days after implantation, when the number of macrophages typically peaked, there were much fewer CD86 positive cells around the Ti@TA‐Sr‐8 implant compared to the Ti group, indicating that TA‐Sr can significantly shorten the inflammatory infiltration process after implantation and advance the healing period. Collectively, these data implied that TA‐Sr endowed the implant with anti‐inflammatory properties and a great potential to manipulate the desired regenerative osteoimmune microenvironment, where M2 macrophages produce growth factors to enhance the differentiation of mesenchymal progenitors and further promote bone healing.

**Figure 7:**
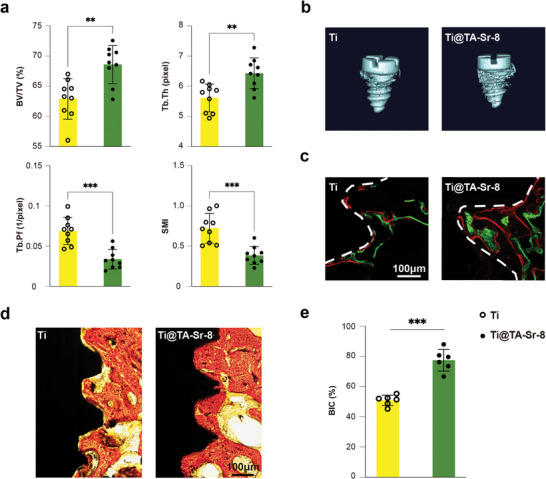
TA‐Sr coatings promoting osseointegration in vivo. a) Micro‐CT analysis of bone regeneration 4 weeks post‐implantation (n = 9); b) Representative 3D reconstructed Micro‐CT images of new bone around different implants; c) Double fluorochrome labeling (green: calcein, week 1; red: alizarin complexone, week 2) at bone‐implant interface (indicated as white dashed line); d) Histological observation of new bone formation at the bone‐implant interface by Van Gieson's staining (red: mineralized bone matrix; yellow: the other tissues; dark: implant); e) quantitative analysis of BIC percentage (n = 6). The data (a,e) were analyzed by two‐tailed unpaired Student's *t*‐test. The error bar represented mean ± SD; ns, no significance, ^**^ *P* < 0.01 and ^***^ *P* < 0.001.

After 4 and 8 weeks of implantation, micro computed tomography (Micro‐CT) was employed to evaluate the bone density (quality) of the surrounding bone. Quantitative analysis at week 4 (**Figure** **7**a) indicated that the percentage of bone volume (bone volume/tissue volume, BV/TV) of Ti@TA‐Sr‐8 was substantially higher than those of the Ti group, which means the amount of bone trabecular is growing and bone anabolism is greater than catabolism. Meanwhile, Ti@TA‐Sr‐8 generated distinct increases in trabecular thickness (Tb.Th) and decreases in trabecular pattern factor (Tb.Pf) and structure model index (SMI), implying the transformation from parallel‐fibered bone to lamellar bone deposition. The augments in both the amount and density of the new bone surrounding Ti@ TA‐Sr‐8 were also confirmed in the images of Micro‐CT reconstruction (Figure 7b). The analysis results for 8‐week postoperative Micro‐CT was shown in Figure S6 (Supporting Information), although there were no significant differences in BV/TV and bone surface to bone volume ratio (BS/BV), better outcomes regarding Tb.Th and connectivity density (Conn. D) were observed in the Ti@TA‐Sr‐8 group compared to the Ti group, indicating a denser and more mature bone mass with greater strength.

Sequential fluorescent labeling was performed to further evaluate the spatiotemporal behavior of new mineral deposition at the bone‐implant interface. Two different fluorochromes were injected intraperitoneally at 1 week (green) and 2 weeks (red) postimplantation. As calcium‐targeting substances, they can preferentially incorporate with the new mineralized tissue, which can be visualized in hard tissue sections by fluorescent imaging. As shown in Figure 7c, the red and green fluorescence bands around TA‐Sr modified implants were significantly wider and larger, and the distances between the two fluorochrome bands were much longer than those around pristine Ti implants, indicating higher efficiency and velocity of mineralization progression on Ti@TA‐Sr. Osseointegration results in ultimate bone‐to‐implant contact (BIC) without an intermediate fibrous tissue.^[^ ^72^ ^]^ Differences in the speed of bone apposition onto the implant surface between the two groups were investigated via undecalcified tissue sections with Van Gieson's staining at week 4, as illustrated in Figure 7d, mineralized bone matrix directly contacted with almost the entire implant surface of Ti@ TA‐Sr‐8 where compact bone prevailed, while the bone surrounding the Ti implant was relatively trabecular, and there was a gap at the bone‐implant interface. Quantitative analysis of BIC percentages (Figure 7e) further revealed that TA‐Sr played an important role in promoting bone‐implant osseointegration at earlier time points after implant placement.

In the context of bone regeneration, BMSCs are considered essential due to their direct role in the mechanisms of repair.^[^ ^73^ ^]^ Osteoblasts originate from committed osteoprogenitor BMSCs and eventually become entrapped in the bone matrix, where they differentiate into osteocytes. When an implant is placed, BMSCs in the vicinity are among the first cells to interact with the new surface. Their attachment, proliferation, and subsequent differentiation on the implant surface are critical determinants of successful osseointegration. TA‐Sr could significantly promote the initial adhesion, recruitment, and osteogenic differentiation of BMSCs, which is bound to further facilitate bone formation.

Numerous studies demonstrate the influence of Sr^2+^ on accelerating osteogenesis and augmenting osseointegration, the mechanisms involved in the activation of Wnt/β‐catenin, CaSR/PI3K/AKT, and NFATc/Maf pathways, which contribute to the bone anabolism in the initial stage.^[^ ^32^, ^74^, ^75^, ^76^ ^]^ On the other hand, TA can selectively inhibit osteoclast proliferation and differentiation probably via suppressing the RANK/RNAKL/OPG pathway.^[^ ^77^ ^]^ To sum up, due to the synergistic effects between TA and Sr^2+^ of alleviating oxidative damage, smoothly switching macrophages from M1 to M2, and facilitating osteoblast activity, TA‐Sr showed great potential in regulating the osteoimmune environment to a much more favorable condition so as to enhance osseointegration. For most implant brands on the market, it always takes about three months for bone to heal after implantation, and even longer when confronted with poor bone quality or compromised health. However, it can be predicted that the use of TA‐Sr‐modified implants will achieve a better result and shorten the treatment period.

Furthermore, most of the implants utilized in clinical practice are irregular and complex in shape, nevertheless, this facile implant modification method is able to achieve a homogeneous loading of Sr within a few minutes before surgery. While the surgeon is cutting and drilling, the assistant can prepare the TA‐Sr modified implant simultaneously. This chairside operation won't delay the clinical operation too much, which is conducive to clinical transformation. On the other hand, MPN can further load a variety of therapeutic drugs. By delivering therapeutic drugs through the implant implantation process to meet different needs, prevent and treat common clinical implant‐related complications such as periimplantitis.

## 3 Conclusion

In summary, we developed a facile but efficient metal phenolic nanocoating on Ti substrates by simply repeating the deposition process of TA and Sr^2+^. The obtained TA‐Sr coatings demonstrated excellent antioxidant properties and were able to transform the attached macrophages from M1 to M2 phenotype. In addition, TA‐Sr significantly promoted stem cell recruitment and accelerated osteogenic differentiation. The nanocoating could effectively inhibit the inflammatory response, convert the osteoimmune environment from a pro‐inflammatory to a pro‐healing state, and consequently enhanced osseointegration at the bone‐implant interface in vivo. In general, our results suggest that TA‐Sr is a promising candidate for surface modification of implants to achieve better and faster osseointegration, which can be easily utilized in future clinical applications.

## 4 Experimental Section

### Reagents and Materials

TA, Dexamethasone, Ascorbic acid, *β*‐glycerophosphate, Calcein, and Alizarin Red S were purchased from Sigma–Aldrich (Shanghai, China). Vitamin B_2_, L‐Methionine, NBT, DPPH, FeSO_4_·7H_2_O, and ABTS were purchased from Aladdin Industrial Co. (Shanghai, China). SrCl_2_·6H_2_O was bought from Macklin (Shanghai, China). Salicylic acid and Rhodamine B were purchased from DAMAO (Tianjin, China). Fetal bovine serum (FBS), MEM Alpha Modification (α‐MEM), and DMEM were bought from Biological Industries (Kibbutz Beit Haemek, Israel). Trypsin, penicillin/streptomycin, phosphate‐buffered saline (PBS, PH7.4), DCFH‐DA, and 4% paraformaldehyde (PFA) were purchased from Biosharp (Hefei, China). Calcein‐AM/PI and FITC‐phalloidin were bought from Solarbio Science & Technology (Beijing, China). DAPI was purchased from the Beyotime Institute of Biotechnology (Shanghai, China). All pure Ti plates (diameter, 33 or 14 mm; thickness, 1 mm) and pure titanium implants (diameter, 1.6 mm; length, 2.5 mm) were purchased from Baoji Titanium Industry Co. LTD.

### Preparation of the Coatings

The Ti plates were successively polished with 400, 1000, and 1500 mesh sandpaper, and ultrasonically cleaned in acetone, ethanol, and deionized water for 15 min each. Then the Ti plates were immersed in 7.6 mL deionized water, 200 µL TA solution (16 mg mL^−1^) and 200 µL SrCl_2_ solution (48 mg mL^−1^) sequentially added dropwise while shaking at 75 rpm. The pH value was then adjusted to 7.4 by adding 40 µL NaOH solution (1 mg mL^−1^) dropwise to the mixture. Each step lasts for ≈30 s. Afterward, the Ti plates were rinsed with deionized water to remove the unabsorbed TA or Sr^2+^. The above procedures were repeated, and the samples were termed according to the repetition times, such as Ti@TA‐Sr‐1, Ti@TA‐Sr‐4, and Ti@TA‐Sr‐8.

### Material Characterization

The surface morphologies of the samples were analyzed by SEM (Gemini SEM 500, Carl Zeiss, Germany). By depositing TA‐Sr on smooth Ti foils instead of Ti plates, the nanoroughness and thickness of the coatings were evaluated using AFM (BioScope Resolve, Bruker, USA). The coordination between TA and metals was confirmed by FTIR (Bruker, Germany). The surface chemical composition and ionic valence were determined by EDS (Pro G5, Phenome, Netherlands) and XPS (ESCALAB250Xi, Thermo Scientific, USA) using an Al Kα source (1201 eV). To investigate the mechanical properties of coatings, nanoindentation measurements were performed using a nanomechanical test instrument (Hysitron TI 980, Bruker, USA). The surface hydrophilicity of different Ti samples was evaluated by measuring the water contact angle (WCA) with a drop‐shape analyzer (Zhong Chen, China). The release of Sr^2+^ from Ti@TA‐Sr in PBS at pH 7.4 and pH 6.0 was detected, respectively, by ICP‐ MS (NexION 350X, PerkinElmer, USA). In addition, TA‐Sr modified Ti plates were immersed in HCl at pH 3.0 for 12 h to determine the total amount of deposited Sr^2+^ by ICP‐MS. To confirm the universal coating ability on different Ti substrates, including wire, mesh, and sponge, Rhodamine B (0.1 µm) was added to the reaction solution, a confocal laser scanning microscope (CLSM, LSM900, Zeiss, Germany) was used to observe the coatings.

### ROS Scavenging Assay—H_2_O_2_ Scavenging Assay

The coated Ti plates with a diameter of 33 mm were placed in the bottom of a 6‐well plate and incubated with 0.1 mL of H_2_O_2_ solution (271.6 mm) at 37 °C for 30 min. The residual concentration of H_2_O_2_ was then detected by a hydrogen peroxide detection kit (Institute of Nanjing Jiancheng Bioengineering, China), the OD value at 405 nm was measured using a microplate reader (SPECTROstar Nano, BMG Labtech, Offenburg, Germany). and then the eliminated H_2_O_2_ was calculated.

The ·OH radical‐scavenging effect of TA‐Sr was measured by ABTS. First, 4.3 mL of deionized water, 250 µL of H_2_O_2_ (200 µm), and 200 µL of FeSO_4_·7H_2_O (18 mm) were mixed together. After ultrasonic treatment for 10 min, different Ti plates were incubated with 1 mL of the mixture for 10 min and then 50 µL of ABTS (1 mm) was added. After 5 min, the UV absorbance at 734 nm was used to estimate the antioxidant activity with a UV–vis spectrophotometer (UV‐2600 I, Shimadzu, Japan).

The ·O_2_^−^ radical scavenging capacity of TA‐Sr was investigated by analyzing the NBT photoreduction inhibition ratio. Ti plates were incubated with a mixture containing 1 mL of PBS (pH 7.4), 200 µL of Vitamin B_2_ (20 µm), 125 µL of L‐methionine (12.5 mm) and 150 µL of NBT (75 µm). The UV absorbance at 560 nm was measured after 15 min of ultraviolet irradiation.

The total antioxidant activity of the coatings was measured on the basis of the discoloration of DPPH. Ti plates (diameter, 14 mm) were placed in the 24‐well plate, and 1 mL DPPH/ethanol solution (0.1 mm) was added to each well. After incubation at 37 °C for 1 h in the dark, the OD value at 517 nm of the supernatant was measured.

To further evaluate the intracellular ROS scavenging capacity of the coatings, RAW 264.7 macrophages were used, which were obtained from the Shanghai Cell Bank of the Chinese Academy of Sciences and cultured in DMEM containing 10% FBS and detached by gently blowing. After sterilizing with 75% alcohol and ultraviolet light for 30 min on each side, Ti plates were placed in 24‐well plates inoculated with 5 × 10^4^ RAW 264.7 cells per well, cultured for 12 h, and then stimulated with 100 ng mL^−1^ lipopolysaccharide (LPS). Six hour later, the cells were stained with 10 µm DCFH‐DA for 30 min and 5 µm MitoSOX Red for 10 min at 37 °C in the dark, respectively, and further observed under a fluorescence microscope (Leica, Wetzlar, Germany).

### Cell Culture

In this study, bone marrow mesenchymal stem cells (BMSCs) were derived from the lower limbs of rats with the approval of the Medical Ethics Committee of Stomatological Hospital of Shandong University (No. 20211001). All measures were taken to minimize pain or discomfort. Freshly isolated BMSCs were cultured in α‐MEM medium containing 1% penicillin‐streptomycin and 20% FBS at 37 °C with a 5% CO_2_ humidified atmosphere. The medium was refreshed every 3 days with *α*‐MEM supplemented with 10% FBS and cells were dissociated by trypsinization when the density reached 80–90% confluence. BMSCs of passages 3 to 5 were used in the subsequent experiments. Murine preosteoblast cells (MC3T3‐E1) were also used, and the relevant experimental procedures were listed in the Supporting Information.

### Biocompatibility Evaluation In Vitro

Sterilized Ti plates were placed in a 24‐well plate. Each well was inoculated with 2 × 10^4^ BMSCs and cells inoculated on tissue culture plastic were used as NC. Cell viability on different substrates was detected by live/dead staining. After 24 h of culture, the cells were stained with Calcein‐AM (2 µm) and PI (4.5 µm), and observed under a fluorescence microscope (Leica). Cell morphology on different samples was observed by SEM (Pro G5, Phenome) after fixation with Gluta fixative (Solarbio), gradual dehydration, lyophilization, and gold sputtering. Furthermore, FITC‐phalloidin was used to mark F‐actin to observe the cytoskeleton. Briefly, after 24 h of incubation, cells were fixed with 4% PFA for 10 min and permeabilized with 0.1% Triton X‐100 in PBS 3 times for 5 min each. Subsequently, cells were stained with FITC‐phalloidin (1:100 diluted) plus DAPI (5 µg mL^−1^) and subjected to a fluorescence microscope (Leica). Additionally, cell proliferation was evaluated on 1, 2, and 3 days with the Cell Counting Kit‐8 (CCK‐8, Dojindo, Tokyo, Japan) according to the procedures provided by the manufacturer.

### Cell Migration Assay

Cell migration assays were performed using Falcon permeable chambers (Corning Inc., Corning, NY, USA) for 24‐well plates. Ti plates of different groups were placed beneath the chamber, in which 200 µL cell suspension (2.5 × 10^5^ mL^−1^ in serum‐free α‐MEM) was added. All chambers were immersed in 500 µL α‐MEM containing 0.1% FBS, except for the positive control (PC) group, which was incubated with 500 µL α‐MEM containing 10% FBS. After incubation for 20 h, cells were fixed with 4% PFA for 15 min at room temperature, then the chambers were rinsed in PBS and stained with 0.1% crystal violet for 5 min. Then the upper surface of the microporous membrane was cleansed with several cotton sticks to remove the cells that had not passed through the membrane. The cells on the bottom side of the membrane were observed under an inverted microscope (Olympus, Tokyo, Japan) and counted by Image J.

### Osteogenic Potential Evaluation

BMSCs were seeded on various Ti plates and incubated for 24 h, then the medium was replaced with osteogenic inducing medium consisting of α‐MEM supplemented with 10% FBS, 10^−8^ m dexamethasone, 50 mg L^−1^ ascorbic acid, and 10^ ^mm β‐glycerophosphate, and the medium was updated every 3 days.

### Osteogenic Potential Evaluation—Quantitative real‐time polymerase chain reaction (qRT‐PCR)

After 7, 14, and 21 days of osteogenic induction, total RNA was extracted and quantitative gene expression analysis of *ALP*, *OCN*, and *Runx2* was carried out by qRT‐PCR. The primer sequences for those genes mentioned above and GAPDH were listed in Table S1 (Supporting Information).

### Osteogenic Potential Evaluation—ALP staining and ALP activity

7 days after osteogenic induction, samples were rinsed with PBS, fixed with 4% PFA for 10 min, and then stained by a BCIP/NBT Alkaline Phosphatase Color Development Kit (Beyotime) in the dark following the manufacturer's protocol. 5 min later, the chromogenic reaction was terminated by washing with deionized water, and the dyeing results were captured by a scanner (FlieScan 1520, Microtek). ALP activity was analyzed with an AKP Detection Kit (Nanjing Jiancheng) and a BCA Protein Assay Kit (Solarbio) according to the manufacturer's instructions.

### Osteogenic Potential Evaluation—ARS Staining

After 14 days of induction, BMSCs were washed with PBS and fixed with 4% PFA for 30 min. Subsequently, the samples were stained with 2% Alizarin Red S (Sigma Aldrich) for 30 min and the images were captured using the scanner as well. Then the quantitative analysis of ARS was achieved by dissolving with 10% cetylpyridnium chloride (CPC, Solarbio), and the absorbance was measured at a wavelength of 562 nm.

### Regulation of Macrophage Polarization

Different Ti plates were fixed in a 6‐well plate, and 2 × 10^5^ RAW 264.7 were seeded into each well. After incubation for 1 and 3 days, total RNA was extracted with a RaPure Total RNA Micro Kit (Magen, China) and reverse transcribed into cDNA using the Evo M‐MLV RT Kit (Accurate Biology, China) following the manufacturer's instructions. qRT‐PCR was performed with the SYBR Green Premix Pro Taq HS qPCR Kit (Accurate Biology, China) on a LightCycler 96 real‐time PCR system (Roche, Basel, Switzerland). *CD11c*, *CD86*, and *IL‐1β* were used as markers of macrophages M1, while *CD206*, *TGF‐β*, and *IL‐10* as markers of M2. The primer sequences for the genes mentioned above and GAPDH were listed in Table S2 (Supporting Information). The protein levels of IL‐1*β* and IL‐10 in supernatants were measured using ELISA Kits (Biolegend, USA) according to the manufacturer's instructions.

Immunofluorescence staining was also adopted to evaluate macrophage polarization. Briefly, RAW 264.7 was cultured on different Ti plates for 24 h at a density of 5 × 10^4^ cells per well in a 24‐well plate, and then fixed in 4% PFA for 15 min and permeabilized with 0.1% TritonX‐100 for 20 min. Subsequently, the samples were further incubated with anti‐CD86 (5.22 µg mL^−1^, CQA1883, Cohesion), anti‐iNOS (4.446 µg mL^−1^, ab178945, Abcam), and anti‐CD206 (2 µg mL^−1^, ab64693, Abcam) at 4 °C overnight, followed by incubation with the corresponding fluorescently labeled Goat Anti‐Rabbit secondary antibody (CD86: CoraLite 488, iNOS: CoraLite 594, CD206: CoraLite 488; 1:500; Proteintech) for 1 h and DAPI for 5 min in the dark. The images were photographed under a fluorescence microscope (Leica), and the fluorescent intensity was quantified and measured by Image J.

### In Vivo Animal Surgical Procedure

All animal experiments were approved by the Medical Ethics Committee of Stomatological Hospital of Shandong University (No. 20211001), and conducted following the guidelines of the Care and Use of Laboratory Animals of the Chinese Science and Technology Ministry. A total of 36 Wistar rats (male, 8 weeks) purchased from Vital River Co. Ltd (Beijing, China) were used in this study, the rats were maintained in a specific pathogen‐free (SPF) animal facility with a 12 h light/dark cycle and regular food and water at 25 °C with air humidity at 55%. All measures were taken to minimize pain or discomfort.

A distal femur metaphysis model was established. Briefly, after anesthesia by intraperitoneal injection of sodium pentobarbital (40 mg kg^−1^), the hind limbs of rats were shaved and sterilized. A 2 cm full‐thickness incision was made on the anteromedial portion of the bilateral femurs to expose the femoral condyles. The implant site was created using a 1.5 mm diameter drill with constant irrigation with 0.9% sterile saline. Each rat was implanted with a Ti control implant and a TA‐Sr modified Ti implant (1.6 mm in diameter, 2.5 mm in length). After surgery, all rats were injected intramuscularly with penicillin (200 000 U mL^−1^) for three consecutive days.

To observe the process of new bone formation and bone remodeling dynamics around the implant, a double‐labeling method was used. The rats received an intraperitoneal injection with calcein (10 mg kg^−1^) 1 week after surgery and alizarin complexone (30 mg kg^−1^) 2 weeks later.^[^ ^78^ ^]^ Rats were sacrificed at specific time intervals (4 and 7 days, 4 and 8 weeks) postoperatively and the femurs containing implants were harvested and fixed in 4% PFA for 24 h.

### Microcomputer Tomography (Micro‐CT)

The specimens collected at week 4 and 8 were scanned with a high‐resolution micro‐CT (Quantum GX, PerkinElmer, Baesweiler, Germany). The Cu 0.06 + Al 0.5 mm filter of X‐ray was selected, and the scanning parameters were set at 90 kV and 88 µA, with 14 min of exposure time and 9 µm of voxel resolution. Raw images were reconstructed and analyzed using CT vox and CT analysis software. The bone tissue around the implants was detected and the region of interest (ROI) included a ring radius 500 µm from the implant surface. Several bone trabecular parameters of ROI, such as BV/TV, BS/BV, Tb.Th, Tb.Pf, Conn. D, and SMI, were analyzed and calculated to assess bone regeneration.

### Immunofluorescence Analysis

All specimens were decalcified in 10% disodium Ethylenediamine tetraacetic acid (EDTA‐Na2, Solarbio, Beijing, China) at room temperature for ≈30 days. Subsequently, the decalcified samples were longitudinally embedded in paraffin wax and cut into 5 µm sections.

In order to detect phenotype switching of macrophages in vivo, immunofluorescence staining was performed. Tissue sections of 4 and 7 days were incubated with primary antibodies of anti‐CD86 (2 µg mL^−1,^ NBP2‐25208, Novus) and anti‐CD206 (10 µg mL^−1^, ab64693, Abcam) at 4 °C overnight followed by incubation with the corresponding fluorescently labeled secondary antibody (CD86: CoraLite594‐conjugated Goat Anti‐Mouse IgG, CD206: CoraLite488‐conjugated Goat Anti‐Rabbit IgG; 1:200; Proteintech) and DAPI. The fluorescent images were obtained with a fluorescence microscope (Olympus), Image J was used to quantify the fluorescence intensity of the stained markers and normalized to DAPI‐stained nuclei counts.

### Hard Tissue Slices and Staining

Bone implant samples of 2 and 4 weeks were embedded in methyl methacrylate after gradual dehydration. Thick sections (30 µm) parallel to the long axis of the implant were cut with a grinder (E400CS; EXAKT Vertriebs Gmbh). After polishing, the fluorescently labeled sections were imaged under CLSM (LSM900, Zeiss) to assess the dynamic osteogenesis process. Then the slices were stained with Van Gieson's (VG) staining and photographed with an inverted microscope (Olympus), and the bone‐implant combination ratio (BIC%) was calculated with Image J.

### Statistical Analysis

All values were presented as mean ± standard deviation (SD). All experiments were repeated at least three times. Statistical analysis was performed with GraphPad Prism 9 (GraphPad Software, USA) using Student's *t*‐test and one‐way ANOVA. Differences were considered to be significant if *P* value was less than 0.05.

## Conflict of Interest

The authors declare no conflict of interest.

## Supporting information

Supporting Information

## Data Availability

Research data are not shared.
