# Supplementary material for: A Multifunctional Metal–Phenolic Nanocoating on Bone Implants for Enhanced Osseointegration via Early Immunomodulation
Source: Adv Sci (Weinh). 2024 Mar 6;11(18):2307269. doi: 10.1002/advs.202307269 (PMC11095205; doi:10.1002/advs.202307269)
Supplement: Supplementary file 1 — Supporting Information [file ADVS-11-2307269-s001.pdf]

ADVANCED SCIENCE
Open Access

Supporting Information

for *Adv. Sci.*, DOI 10.1002/advs.202307269

A Multifunctional Metal–Phenolic Nanocoating on Bone Implants for Enhanced Osseointegration via Early Immunomodulation

Jin Liu, Yilin Shi, Yajun Zhao, Yue Liu, Xiaoru Yang, Kai Li, Weiwei Zhao, Jianmin Han, Jianhua Li* and Shaohua Ge**

Supporting information

TABLE S1 Primer sets (Rattus) used for quantitative real-time PCR.

RNA template	Forward primer (5′-3′)	Reverse primer (5′-3′)
GAPDH	TCTCTGCTCCTCCCTGTTCT	ATCCGTTCACACCGACCTTC
ALP	ACAACACCAACGCTCAGGTC	GTGACCTCGTTCCCCTGAGT
OCN	TGACAAAGCCTTCATGTCCAAG	GAAGCCAATGTGGTCCGCTA
Runx2	ACGAATGCACTATCCAGCCA	GCAGGTACGTGTGGTAGTGA

TABLE S2 Primer sets (Mice) used for quantitative real-time PCR.

RNA template	Forward primer (5′-3′)	Reverse primer (5′-3′)
GAPDH	TGTCTCCTGCGACTTCAACA	GGTGGTCCAGGGTTTCTTACT
CD11c	TCTTCTGCTGTTGGGGTTTGT	TCAGCACCGTCCATGTGAAA
CD86	TCTGCCGTGCCCATTTACAA	TGTGCCCAAATAGTGCTCGT
CD206	GCTTCCGTCACCCTGTATGC	CTGCTCCACAATCCCGAACC
IL-1β	GTGTCTTTCCCGTGGACCTT	AATGGGAACGTCACACACCA
IL-10	CACTACCAAAGCCACAAGGCA	GAGCAGGCAGCATAGCAGTG
TGF-β	GCAACAATTCCTGGCGATACC	ATTTCCCCTCCACGGCTCAA
OCN	TCTGACCTCACAGATGCCAAG	AGGGTTAAGCTCACACTGCT
ALP	GCACCTGCCTTACCAACTCT	GTGGAGACGCCCATACCATC
Col-1	CCCTGGTCCCTCTGGAAATG	GGACCTTTGCCCCCTTCTTT
Runx2	CAGGCAGTTCCCAAGCATTT	GGTAAAGGTGGCTGGGTAGT

TABLE S3 Elemental analysis of different substrates.

Element	Weight percentage (%)			
	Ti	Ti@TA-Sr-1	Ti@TA-Sr-4	Ti@TA-Sr-8
Ti	92.94	92.63	91.67	90.64
O	6.19	5.87	6.38	6.81
C	0.88	0.9	0.95	1.06
Sr	0	0.61	1.01	1.48

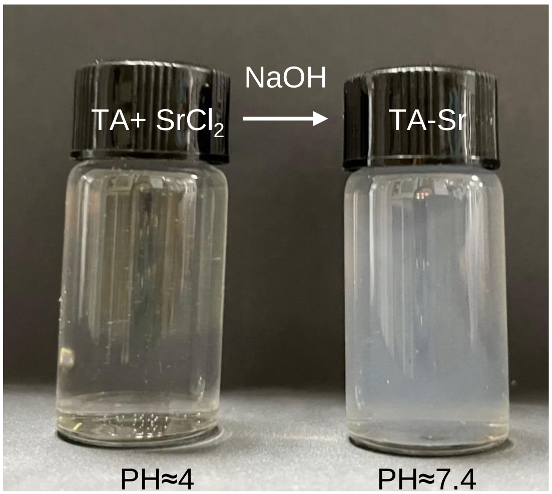

Figure S1. Photograph of changes in the reaction solution.

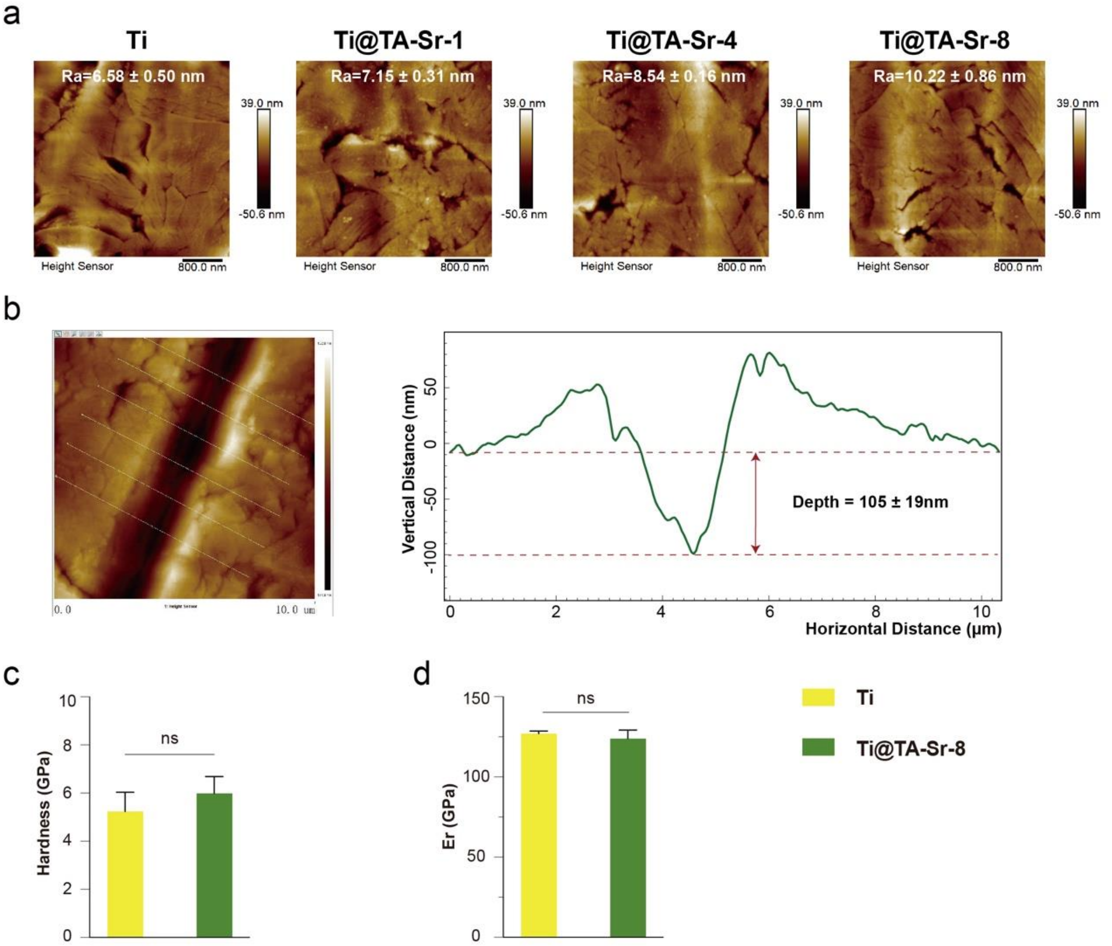

Figure S2. (a) Surface morphologies and roughness of the coatings deposited on smooth Ti foils observed by AFM (n=3); (b) Thickness of TA-Sr-8 evaluated by scratch test (n=6); (c, d) Hardness and reduced modulus (Er) of coated and uncoated Ti substrates (n=5), analyzed by two-tailed unpaired Student's *t*-test. The error bar represented mean ± SD; ns, no significance.

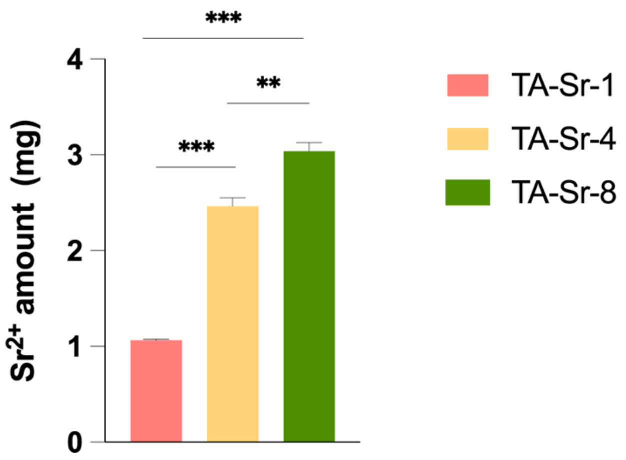

Figure S3. Total amount of Sr^{2+} detected by ICP-MS (n=3). The data were analyzed by one-way ANOVA test. The error bar represented mean ± SD; $^{**}P < 0.01$ and $^{***}P < 0.001$.

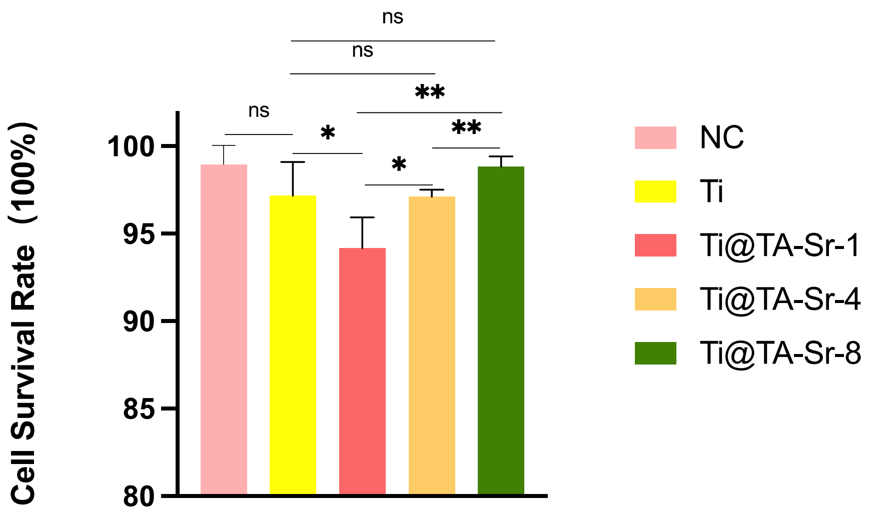

Figure S4. Percentages of live cells of BMSCs cultured on coated or uncoated Ti plates（n=5）. The data were analyzed by one-way ANOVA test. The error bar represented mean ± SD; ns, no significance, $^{*}P < 0.05$, $^{**}P < 0.01$.

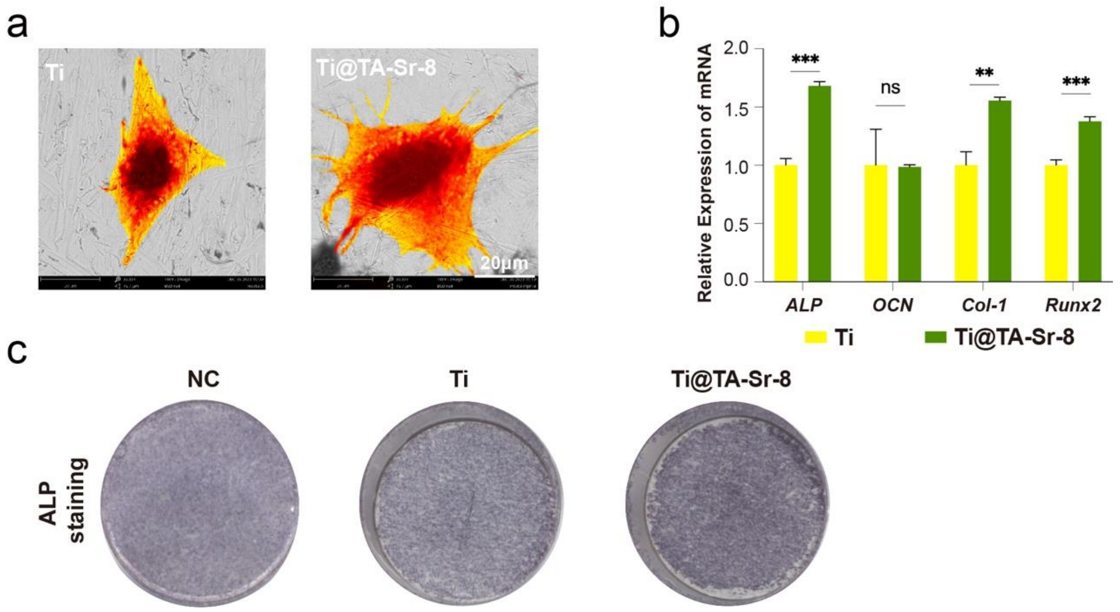

Figure S5 (a) Morphologies of MC3T3-E1 after 24 h of culture captured by SEM (cells were pseudocolored orange for visual observation); (b) osteogenesis-related genes relative expression of *ALP*, *OCN*, Col-1, and *Runx2* detected qRT-PCR analysis (n=3); (c) Representative photographs of ALP staining of MC3T3-E1 in different groups at day 7. The data (b) were analyzed by two-tailed unpaired Student's *t*-test. The error bar represented mean ± SD; ns, no significance, $^{**}P < 0.01$ and $^{***}P < 0.001$.

MC3T3-E1 were cultured in α-MEM medium containing 1% penicillin-streptomycin and 10% FBS at 37°C with a 5% CO_2 humidified atmosphere. The medium was refreshed every 3 days and the cells were dissociated by trypsinization when the density reached 80-90% confluence. After incubating for 24h, the cell morphologies on different samples were observed by SEM (Pro G5, Phenome) after fixation with Gluta fixative (Solarbio), gradual dehydration, lyophilization, and gold sputtering.

MC3T3-E1 cells were seeded on various Ti plates and incubated for 24 h, then the medium was replaced with osteogenic inducing medium consisting of α-MEM supplemented with 10%FBS, 10^{-8} M dexamethasone, 50 mg/L ascorbic acid, and 10 mM β-glycerophosphate, and the medium was updated every 3 days.

Quantitative real-time polymerase chain reaction (qRT-PCR): After 7 days of osteogenic induction, total RNA was extracted and quantitative gene expression analysis of *ALP*, *Col-1, OCN* and *Runx2* was carried out by qRT-PCR. The primer sequences for those genes mentioned above and GAPDH were listed in Table S2.

ALP staining: After 7 days of osteogenic induction, samples were rinsed with PBS, fixed with 4% PFA for 10 min, and then stained by a BCIP/NBT Alkaline Phosphatase Color Development Kit (Beyotime) in the dark following the manufacturer's protocol. 5 min later, the chromogenic reaction was terminated by washing with deionized water, and the dyeing results were captured by a scanner (FlieScan 1520, Microtek). ALP

activity was analyzed with an AKP Detection Kit (Nanjing Jiancheng) and a BCA Protein Assay Kit (Solarbio) according to the manufacturer's instructions.

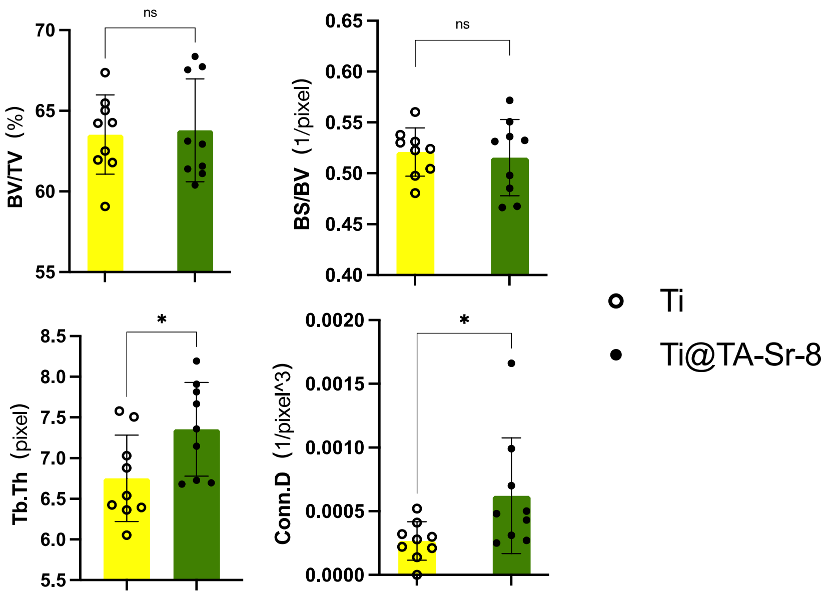

Figure S6. Micro-CT analysis of bone regeneration 8 weeks post-implantation (n=9). The data were analyzed by two-tailed unpaired Student's t-test. The error bar represented mean ± SD; ns, no significance, $^{*}P < 0.05$.
